# Implementation of IFPTML Computational Models in Drug Discovery Against Flaviviridae Family

**DOI:** 10.1021/acs.jcim.3c01796

**Published:** 2024-03-11

**Authors:** Yendrek Velásquez-López, Andrea Ruiz-Escudero, Sonia Arrasate, Humberto González-Díaz

**Affiliations:** † Departamento de Química Orgánica e Inorgánica, Facultad de Ciencia y Tecnología, Universidad del País Vasco/Euskal Herriko Unibertsitatea UPV/EHU. Apdo. 644. 48080 Bilbao (Spain); ‡ Bio-Cheminformatics Research Group, Universidad de Las Américas, Quito 170504, (Ecuador); § Department of Pharmacology, University of the Basque Country UPV/EHU, 48940 Leioa, (Spain); ∥ IKERDATA S.L., ZITEK, University of Basque Country UPV/EHU, Rectorate Building, 48940 Leioa, Spain; ⊥ BIOFISIKA, Basque Center for Biophysics CSIC-UPV/EHU, 48940 Bilbao (Spain); # IKERBASQUE, Basque Foundation for Science, 48011 Bilbao (Spain)

## Abstract

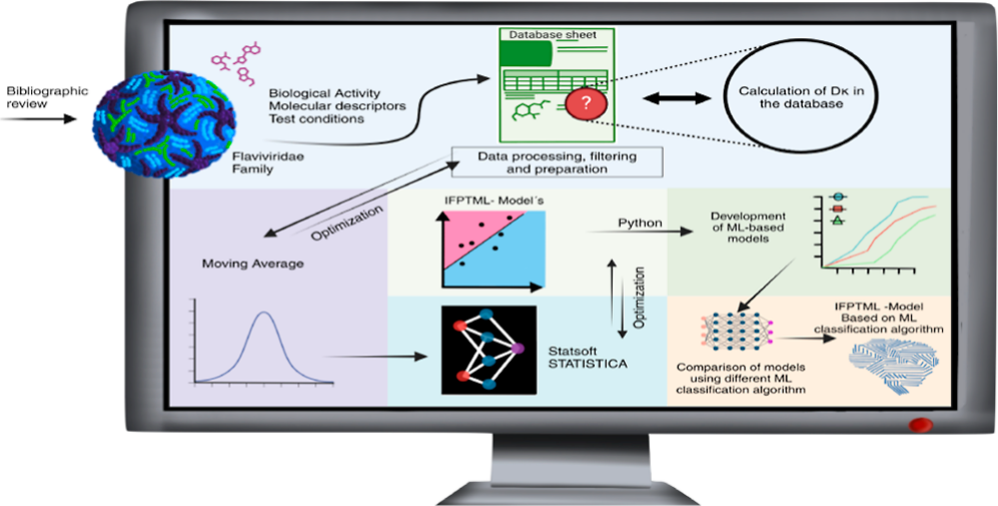

The Flaviviridae family consists of single-stranded positive-sense RNA viruses, which contains the genera *Flavivirus*, *Hepacivirus*, *Pegivirus*, and *Pestivirus*. Currently, there is an outbreak of viral diseases caused by this family affecting millions of people worldwide, leading to significant morbidity and mortality rates. Advances in computational chemistry have greatly facilitated the discovery of novel drugs and treatments for diseases associated with this family. Chemoinformatic techniques, such as the perturbation theory machine learning method, have played a crucial role in developing new approaches based on ML models that can effectively aid drug discovery. The IFPTML models have shown its capability to handle, classify, and process large data sets with high specificity. The results obtained from different models indicates that this methodology is proficient in processing the data, resulting in a reduction of the false positive rate by 4.25%, along with an accuracy of 83% and reliability of 92%. These values suggest that the model can serve as a computational tool in assisting drug discovery efforts and the development of new treatments against Flaviviridae family diseases.

## 1 Introduction

Arboviruses (ARthropod-BOrne VIRUSes) are organisms transmitted by hematophagous arthropods.^1^ Within this group, we find the Chikungunya virus (CHIKV), West Nile Virus (WNV), dengue virus (DENV), yellow fever virus (YFV), and Zika virus (ZIKV), transmitted by mosquitoes of the *Aedes* spp. genus “*Aedes Aegypti*”.^2^ Generally, members of this genus, especially those who are transmitted by the same vector, share a similar symptomatology but approximately 80% of infections are asymptomatic.^3^ Although there are variations, such as in Zika that present a unique illness (Guillain–Barré syndrome and Microcephaly in neonates).^4^

Taxonomic analyses have classified DENV, CHIKV, YFV, and ZIKV as members of the Flaviviridae family, *Flavivirus* genus.^5−7^ This genus is characterized by a positive-sense RNA of approximately 11 kb.^8,9^ It has been reported that the proteins encoded by RNA are conserved in members of the *Flavivirus* genus, for example, DENV serotype 4 shows high similarity to its counterparts in the genus transmitted by mosquitoes.^10,11^

Currently, the development of vaccines and drugs against certain members of the *Flavivirus* genus (YFV, DENV, WNV, Japanese Encephalitis Virus—JEV, etc.) is more widespread than against ZIKV.^12^ Due to the highly conserved genome among them and ZIKV, the same treatments and medications have been used to treat this disease hopping that they will have the same success among the other members of their family.^12−14^ However, the development of these medications has not managed to surpass the second phase of drug development.^15,16^

In order to manage the dilemma, the development of computational chemistry has favored the discovery and design of novel drugs.^17−20^ Currently, the identification and optimization of chemical compounds as potential candidates for pharmaceutical targets of interest have been investigated.^21^ The development of computational models based on machine learning (ML) is a widely used technique in computer-aided drug design.^22^ These models use the structural information on compounds and established assay conditions to elucidate new compounds that can interact with the desired targets.^23,24^

With the rise of Big Data and the advent of the digital era, a large amount of structural information about proteins and small molecules has been obtained.^25^ This information could provide researchers and experimental developers with new pharmacological targets to be tested, opening a range of possibilities and opportunities for these methods to be used for drug discovery based on learning techniques.^26^ The amount of data that can be obtained from these new methods and techniques can provide insights into the intrinsic relationships that could enable the generation of prediction models to encode chemical structures. These new techniques are based on experimental and computational information; therefore, can be used to predict the activity of new compounds of pharmaceutical interest.

By developing ML models, new systems can be created with the ability to learn and improve without undergoing any previous programming.^27−29^ The accuracy and effectiveness of these models must meet criteria of responsibility, such as systematic identification and search for regularities to guarantee the prediction and ensure that the analysis was carried out correctly.^30^

These kinds of methods have been used in other scientific fields such as robotics, data mining, chemistry, and biotechnology, etc.^31−33^ However, one of the limitations of these conventional methods arises from the fact that they don’t cover large data sets that appear in trials. Using the clinical data available as an example, there is useful data such as cells, proteins, and organisms that were used in the trials, and these extra data can be used to enrich these models.^34^

Perturbation Theory Machine Learning (PTML) models were developed to solve this problem, taking perturbation theories (PT) combined with ML models to obtain PTML models.^24^ They are based on a reference property *f*(*v*_*ij*_)_ref_.^35^ Perturbation operators PT are added to this property to measure the deviations that can occur with respect to this reference property; in this way, it can predict the properties of an unknown system but similar to the original system that was used as a refs (36–38).

It has been shown that PTML models are applicable to various correlation problems. However, most applications of these methods have focused on classification problems. PTML methods present the calculated function and show it relating it to the membership of a system to different classes.^35^ These systems can have different values of *v*_*ij*_ = biological activity in an *n*th system under multiple *c*_*j*_ = test conditions. This can be extended to multiple parameters within *v*_*ij*_ that have been measured in various ***c***_***j***_ assays. These parameters can be optimized.^21^ As mentioned earlier, the model takes *f*(*v*_*ij*_)_ref_ = *p*(*v*_*ij*_/*c*_*j*_)_ref_ as input data, which represents the probability that other similar systems take favorable values *f*(*v*_*ij*_)_obs_ 1 in clinical trials that usually have the same conditions *c*_*j*_.^35^

Currently, there is an outbreak of new viral diseases, and also new variants emerging from previously reported disease. *Flaviviruses*, in particular, have been a major problem since the last century and continue to be one of the leading causes of death in South America.^39^ Although compounds that can prevent the replication of these viruses have been synthesized, the variability of this family makes them a challenge for researchers and public health. Nowadays, the DENV has four different serotypes. In 2016, the ZIKV was classified as a severe disease due to of its effects on newborns.^10,40^ Most viruses in this family have similar structures; however, there is no specific drug to treat any of them.^9,41^ Therefore, it is necessary to accelerate the methods of drug discovery and development. The traditional approach involves synthesizing compounds and testing them, which is a trial-and-error process.^42,43^ The use of chemoinformatic techniques, such as the PTML method, can accelerate the discovery of potential drug candidates that can inhibit the development of this type of virus.

**Figure 1:**
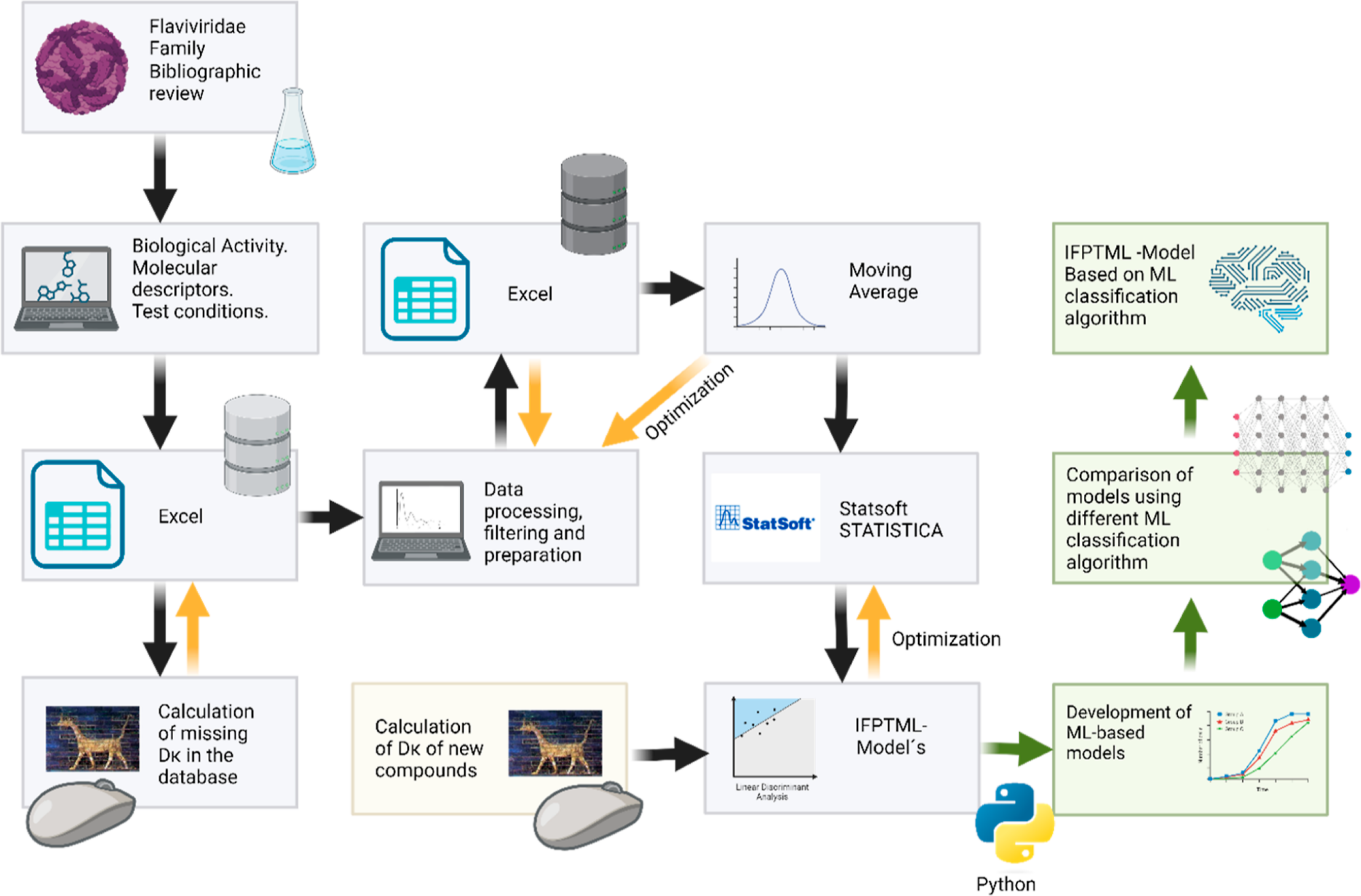
General workflow.

The general flowchart showing the interconnections between the different parts of this work: (1) chemoinformatic study, (2) statistical analysis and (3) biological assays probability, is depicted in Figure 1.

## 2 Materials and Methods

### 2.1 Data Preparation and Processing

#### 2.1.1 Data Set Generation

The generation of the data set or sample to be analyzed consist on the search and compilation of compounds tested in certified databases. First, it is necessary to determine the target or underlying disease to create the model. The members of the Flaviviridae family transmitted by the tropical vector “*Aedes aegypti*” were considered for the development of the data set. The ZIKV was selected as the base due to previous studies conducted with this virus, which provided a curated database. However, because it is a disease in developing countries, the existing data is not very significant in terms of quantity. The data required to train a model is much larger than the available; therefore, this database was expanded to include other members of the family with the genetic similarity (>90%) that are transmitted by mosquitoes. Using the ChEMBL database,^44^ a search was made based on compounds that previously reported activity against selected targets (ZIKV, DENV, DENV2, DENV3, DENV4, HepC, WNV, YFV, etc.). The large data set obtained should report the biological activity of the assays as data. Subsequently, this data set was sorted using Office360. The resulting data set included 47,405 compounds with biological activity. The reported activity is detailed in different measurement forms such as IC_50_ (nM), *K*_i_ (nM), inhibition (%), potency (nM), activity (%), etc. (see Supporting Information file DATASETS.xlsx for details of assay conditions).

A preparation of the collected data must be carried out within the PTML-based model. Molecular descriptors are used as part of its equation, with the most used ones in this type of classification models being D_1_: MW, D_2_: ALogP, and D_3_: TPSA. These values are easy to calculate and are present in the literature, providing reliable information. As for the assay conditions, they are obtained during the generation of the database because they represent the conditions under which the assays were performed. The assay conditions include C_1_: target name, C_2_: target organism, C_3_: assay organism, C_4_: assay tissue name, C_5_: assay cell type, and C_6_: subcellular assay. Primarily, these conditions can be modified, or additional conditions can be added to improve the model or consider specific conditions desired in an assay. In this study, three molecular descriptors and six assay conditions were used in the model development. Subsequently, this selection was expanded to include thirty-two descriptors under the six reported assay conditions. The descriptors were obtained using the Dragon software, which is widely used and user-friendly according to the literature. It is necessary to calculate the molecular descriptors of these compounds using the DRAGON software.^45^ Thus, the descriptors were obtained to complete the necessary information for the model to analyze.

#### 2.1.2 Post Processing

The processing of data is a crucial step in PTML models. It utilizes molecular descriptors *D*_*k*_, as well as their deviation against the expected value of the reference system. The reference value of a system is measured as the average descriptor value for each assay found in the database under conditions ⟨*D*_*k*_ (***c***_***j***_)⟩. The model itself takes into account the descriptor values and the assay conditions in which they were performed (where and how?). The problem that arises from using all this information lies not in the numerical values but in the nominal variables that may be encountered. Therefore, it is necessary to include a new variable that can accept these nominal variables and interpret them numerically. The homogenization of moving average (MA) as a statistical tool is used to analyze ordered sets, thereby eliminating the randomness present in the data. Within this tool, the chosen conditions directly influence the MAs. An equation is proposed that considers a variety of conditions simultaneously, resulting in a multiple MA. Under this premise, the eq 1 was utilized.
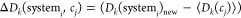
1Being ⟨*D*_*k*_ (***c***_***j***_)⟩ the average of the values presents in the variables *D*_1_,*D*_2_...*D*_*n*_, where *D* represents the molecular descriptors. The assay conditions are represented by ⟨***c***_***j***_⟩ where *j* = 1,...,*n*. Finally, *D*_*k*_(system_*i*_)_new_ represents the *D*_*k*_ values of the compounds found in the database. With the data mentioned in the equation ⟨MA⟩, it seeks to measure how much the descriptors of an assay deviate from the average under specific conditions ***c***_***j***_.

The expected outcome of this model is for it to be capable of predicting the experimental value of  for the compounds in the database. Similarly, the model will be able to determine the activity value of unknown compounds . In this way, the variable  is defined as a value measured from the reported biological activity, which takes into account the assay conditions directly associated with the target diseases.

Due to the variability of the units present in the databases, it is not possible to consider them immediately. This is why a transformation of these values must be performed in order to classify them. A new parameter *d*(*c*_0_) was established, which decides whether a value is desirable or undesirable. If *d*(***c***_**0**_) = 1, indicates that an increase in the analyzed parameter will be desirable, while *d*(***c***_**0**_) = −1 indicates that a decrease in the parameter will be undesirable. The observed function must establish a limit or cutoff point to define whether a unit is desirable or not. Concentrations are set as −1, percentages and activities = 1, and effectiveness and speed = 1. In cases where the cutoff points are not defined, *v*_*ij*_ > 1000 is established. If these conditions are not met, the average activity ⟨*v*_*ij*_⟩ is used.

In order to calculate the average value ⟨*v*_*ij*_⟩ of compounds that have the same activity measure, values must be established based on whether they are favorable or not. This determination depends on whether their values were above or below the mean value of the data, classifying them into a binary system (active = 1 and inactive = 0). The value of the function *f* (*v*_*ijk*_)_obs_ is defined in this study as an experimental value because its result will be the output variable, based on whether a compound in the database is active or not, providing an idea of their activity. With the previous calculations, the conditions are established as follows.

- If *d*(*c*_0_) = 1 and (*v*_*ijk*_)_obs_ > *cutoff* and/or above the established mean value, *f* (*v*_*ijk*_)_obs_ = 1. Otherwise, *f*(*v*_*ijk*_)_obs_ = 0

Likewise:

- If *d*(*c*_0_) = 1 and (*v*_*ijk*_)_obs_ < *cutoff* and/or below the established mean value, *f* (*v*_*ijk*_)_obs_ = 1. Otherwise, *f*(*v*_*ijk*_)_obs_ = 0.

The function *f* (*v*_*ijk*_)_obs_ will apply the cut-offs as control points, enabling it to predict the activity of a compound based on the condition of (***c***_**0**_). The accuracy of the *f* (*v*_*ijk*_)_obs_ will be defined by the specificity of the classification cutoff, making it crucial to define this limit for the model’s integrity. Finally, it is necessary to define a reference variable with known values that have been previously reported to be active in experimental assays. This function uses probabilities and represents the likelihood of compounds being reported as active under the established condition (***c***_**0**_) and for each sublevel j, see eqs 2 and 3.
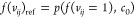
2
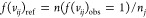
3

### 2.2 Computational Methods

#### 2.2.1 PTML Model Development

The PTML model uses the previously calculated variables in the methodology as input variables. The function *f*(*v*_*ij*_)_ref_, the Δ(*D*_*k*_), and *D*_*x*_ are employed. The output variable *f* (*v*_*ij*_)_exp_, obtained from this model, enables the binary classification (1 and 0). Linear discriminant analysis (LDA) is employed to find a linear combination of these variables, allowing the model to effectively separate the two types of values within a single statistical process.

For data processing, the Statistica 10.0^46^ software was used. Out of the total data, 75% was allocated for training, and the remaining 25% was used for method validation. The resulting statistical parameters (specificity and sensitivity) of the equation obtained should fall between 75 and 95%. A prediction capability below 70% would be insufficient, rendering the model unacceptable. Following the LDA statistical test, the model yields an output variable *f*(*v*_*ij*_)_calc_, where the values from this function correspond to the actual values of predicted activity based on probability. The coefficients of the PTML equation are also obtained from this analysis. Finally, Mahalanobis distances are employed to transform the dimensionless results of the equation into preprobability functions. This enables binary classification and facilitates future predictions for the development or discovery of new compounds.
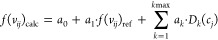
4

#### 2.2.2 ROC Validation Method

A receiver operating characteristic (ROC)^47^ curve was used as a graphical representation to evaluate the screening method. The graph used explains the success and error of the model, the true positive values are placed on the *Y*-axis and the apparent positive values on the *X*-axis. This arrangement allows the analysis of the accuracy of the model. The ROC curve represents the proportion of values that were correctly predicted versus those that were incorrect. This way, by calculating the area under the curve it is possible to get this proportion value, that should be the highest possible value that can be obtained.

#### 2.2.3 Classification ML Models through Python

For the development of ML classification models Python programming language was used together with NumPy, Scikit-learn and PyCaret libraries.

The data set for the training and validation of the model was IFPTML-Flaviviridae *D*_*k*_30. To compare the performances of the previously created model with LDA and the python model, the training and validation subsets remained unchanged. Different models were compared by using the PyCaret classification function “compare_models”. This function trains the algorithms that are available in the library and orders the best models based on their accuracy metric by default. All the performance metrics that are listed in this function are the accuracy, AUCROC, precision, recall, f1-score, Cohen kappa score and Matthews correlation coefficient. The optimization of the model which showed the best overall performance was done using the function “tune_model” to find the optimal hyperparameters. The evaluation of the final model was done with the “evaluate_model” function. This shows a variety of results including the hyperparameters, AUCROC curve, confusion matrix and feature importance, among others.

Accuracy is the rate of the correctly classified cases. Precision measures the fraction of true positives among all the predicted positives. Recall (sensitivity) is the rate of true positives. F1 score is the harmonic mean of recall and precision.^48^ AUCROC is a metric that assesses the ability of the model to discriminate between classes. A perfect model would have an AUCROC value of 1, indicating a perfect classification, while a value of 0.5 suggests random performance, equivalent to chance.^49^ MCC correlates the real and predicted scores in binary classifications considering all the true and false instances.^50^ Cohen’s Kappa is typically used in binary classification problems to assess the agreement between two classifiers using the traditional 2 × 2 confusion matrix.^51^ (see Table 1).

**Table 1:** Formulas of Accuracy, Precision, Recall, F1, MCC and Cohen’s Kappa Performance Metrics^a^

performance metric	formula
accuracy	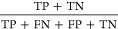
precision	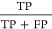
recall	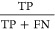
F1	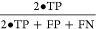
MCC	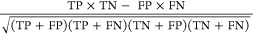
Cohen’s kappa	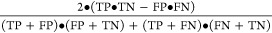

a TP = true positive, TN = true negative, FP = false positive, FN = false negative.

## 3 Results and Discussion

### 3.1 IFPTML-Flaviviridae Model

The construction of a model capable of predicting the probability of a compound being biologically active against a disease, would be a tool that helps reduce costs and time in drug discovery. This method must be reliable and reproducible. The goal of this investigation was to build a classification model based on the Flaviviridae family that has the best statistical parameters and includes variables of interest.

The first result to achieve was proper data cleansing, which is the first checkpoint to get a functional model. This step is essential for the development and correct functioning of the model. The filtering of the 47,382 assays had to be done in a way that allows its use in the construction of the model without causing erroneous results or generating undesired false positives.

The 46,518 resulting assays after cleansing should be ready for analysis. Of these, 10,910 are members of the *Flavivirus* genus, and the rest are members of the Flaviviridae Family. It is expected that the clean data will not affect the calculations that will be performed, as the model learns, trains, and improves with each treatment it receives.

Due to the wide variety of units and measurements found in the databases, these must be standardized to a common measure or unit. As mentioned in the Methods Section, calculations were performed to homogenize the data, resulting in MA values for every *c*_0_. From these results, experimental values and reference values were calculated, as shown in the annexes.

The obtained experimental values *f*(*v*_*ij*_)_obs_ and the reference values *f*(*v*_*ij*_)_ref_, together with the LDA using Statistica 10.0 software, allowed the construction of several models, considering statistical parameters such as specificity (Sp (%) = 0), sensitivity (Sn (%) = 1), and accuracy (Ac (%) = percentage of correct predictions within the analyzed data). To determine whether a parameter is good or not within the constructs, it is established that the minimum values for consideration should be those with specificity, sensitivity, and accuracy values above 75% in both, the training and validation series.

The PTML models start with the input variables *f*(*v*_*ij*_)_obs_, *f*(*v*_*ij*_)_ref_, and Δ*D*_*k*_(***c***_***j***_), to which the effects of the perturbators will be added according to the established parameters and selected variables. The resulting equation takes into account the corresponding operators for all possible cases of *D*_*k*_ = *MW*,*ALogP*, and *TPSA*, and their respective (***c***_***j***_). The expectation is to obtain an equation that covers the greatest number of possible scenarios. Equation 5 presents a PTML-LDA model considering the simple and simplified variables. A Chi-square test was also performed as a classifier between the classes (*f*(*v*_*ij*_)_obs_ = 0 vs *f*(*v*_*ij*_)_ref_ = 1).
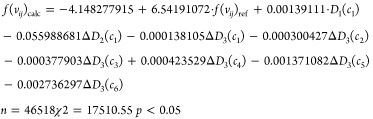
5

The resulting equation was selected after comparing it to several models constructed, using the same input variables but with different effects during the LDA. For a model to be considered optimal, it must contemplate the greatest possible number of conditions in its equation. In statistical analysis, the model can be programmed to figure in all possible effects or to choose the best effects with highest influence. Among the various constructs obtained, there were several that, despite considering all *D*_*k*_, did not reflect the effect of all conditions ***c***_***j***_. These were discarded because they could present undesired results, such as false positives, this occurs when the model is tested with new data and none of the desired conditions are found within the ***c***_***j***_ of the equation. In consequence the model will not consider them, resulting in the loss of information or incomplete results.

The constructs that were obtained must consider the influence of *D*_*k*_, while leaving out the certain conditions ***c***_***j***_. Likewise, constructs that reflect the influence of the conditions ***c***_***j***_ while leaving out the *D*_*k*_, were also obtained. The changes in these constructs are based on correctly establishing the input variables and ensuring that the data is properly homogenized and filtered. The Incorrect settings of the cutoffs can cause an alteration of the final result, leading to more frequent false positives. One of these changes was very significant in an obtained construct, allowing to corroborate the influence of these limits and how their variations can favor or hinder the selection of the model. Many times, it is not possible to obtain an equation that considers all the input variables because there may be ***c***_***j***_ conditions or *D*_*k*_ that cannot be related, so they will be excluded. In these cases, is possible to obtain a viable equation that provides favorable results through a proper data handling. The more specific information obtained, the more accurate the cutoffs are, the more related the data is, and the model can be improved. The selected model as the final result takes into account all these critical points of selection, encompassing the greatest number of ***c***_***j***_ conditions and the *D*_*k*_ in its equation (see eq 5). Table 2 summarizes the results obtained.

**Table 2:** Results of the IFPTML-Flaviviridae-LDA Model Where the Values of Sp, Sn and Ac of the Best Model Obtained are Presented

sets	param.		scope of test *p*(1) = 0.85		
expected stat.		predicted values	***n***_***j***_	***f***(***v***_***ij***_)_**pred**_ = **0**	***f***(***v***_***ij***_)_**pred**_ = **1**
Training Series					
***f***(***v***_***ij***_)_**obs**_ = **0**	Sp (%)	75.95	19,388	14,726	4662
***f***(***v***_***ij***_)_**obs**_ = **1**	Sn (%)	78.88	15,500	3273	12,227
total	Ac (%)	77.25	34,888		
Validation Series					
***f***(***v***_***ij***_)_**obs**_ = **0**	Sp (%)	75.5	6457	4875	1582
***f***(***v***_***ij***_)_**obs**_ = **1**	Sn (%)	79.29	5172	1071	5683
total	Ac (%)	77.19	11,629		

During the development of this method, several constructs were created in order to reach the final model. Twenty analyses were performed for each model using this method. The goal was to find the best model-based on three statistics parameters to satisfy. Since the models must have statistical values higher than 75%,^36^ a balance between specificity, sensitivity, and accuracy was sought. In consequence, the model will be able to correctly predict compounds that have high probabilities of being active and differentiate between active and inactive compounds. It is also mentioned that the training and validation values should be similar to each other, see Table 3 (see Supporting Information file DATASETS.xlsx for details of the data set used and detailed results of the model for each case).

**Table 3:** Results of PTML-LDA Models, the Comparison of Their Values of Sp, Sn and Ac

PTML-LDA models 16 and 24				PTML-LDA model proposed	
training model no. 16	validation series no. 16	training model no. 24	validation series no. 24	training model no. proposed	validation series no. proposed
Sp = 77.63	Sp = 76.88	Sp = 77.42	Sp = 76.71	Sp = 75.95	Sp = 75.50
Sn = 75.88	Sn = 75.97	Sn = 76.06	Sn = 76.18	Sn = 78.88	Sn = 79.29
Ac = 76.85	Ac = 76.47	Ac = 76.81	Ac = 76.47	Ac = 77.25	Ac = 77.19

The tables result present an equitable distribution according to the established parameters. The chi-square test allows us to confirm that the classification groups are divided, and the p-value is under or equal to 0.05 (eqs 5).

Among the various constructs created to determine the best model, several of them included both desirable characteristics (conditions ***c***_***j***_ and *D*_*k*_). However, their statistical parameters were poor, leading their classification as incomplete due to the characteristics they exhibited. The model that presented the best statistical parameters, as well as meeting the criteria ***c***_***j***_ and *D*_*k*_, obtained a precision value of 77%. When comparing the results of the models, it was corroborated that an IFPTML-LDA model was found, and both (its equation and its statistical parameters) met the desired characteristics. In the final part of this results segment, the ROC validation method was employed to verify the model.

### 3.2 A Comparison with Genus *Flavivirus* and Flaviviridae IFPTML Method

During the development of the final IFPTML model, several test models were created. One of them used only the data from members of the *Flavivirus* genus that are transmitted by hematophagous arthropods. These data were processed according to the procedure described in the experimental development, with *D*_*k*_ = 3 and ***c***_***j***_ = 6. The data used for the IFPTML-*Flavivirus* model represented 24% of the total data used in the main model. The statistical parameters showed favorable values in general, but the classification matrix failed as it classified false positives with high significance (see Table 4).

**Table 4:** Results of the IFPTML-*Flaviviruses*-LDA Model, Statistical Parameters Sp, Sn and Ac for Training and Validation

sets	param.		scope of test *p*(1) =		
expected stat.		predicted values	***n***_***j***_	***f***(***v***_***ij***_)_**pred**_ = **0**	***f***(***v***_***ij***_)_**pred**_ = **1**
Training Series					
***f***(***v***_***ij***_)_**obs**_ = **0**	Sp (%)	82.61	6551	5412	1139
***f***(***v***_***ij***_)_**obs**_ = **1**	Sn (%)	79.66	1632	332	1300
total	Ac (%)	82.02	8183		
Validation Series					
***f***(***v***_***ij***_)_**obs**_ = **0**	Sp (%)	80.40	2168	1743	425
***f***(***v***_***ij***_)_**obs**_ = **1**	Sn (%)	75.85	559	135	424
total	Ac (%)	79.46	2727		

The IFPTML-*Flavivirus*-database consists only of members of the *Flavivirus* genus; as mentioned in the introduction, the members are closely related to each other and present conserved regions in their genomes.^10^ One of the most studied *Flaviviruses* is the DENV, and its drugs are used as models to treat other members of this species.^10,12,41^ Therefore, several studies have reported similarities in their compound conditions or characteristics. However, these studies vary in reported activity because of the 10% difference, which makes each of these members unique in their own way.^9,16,40^ Taking this into consideration, the unique characteristics of these variables should be expanded to make them more specific. The false identification of an element can be due to its similarity to others that fit the model (true positives).^52,53^ Therefore, a new model was proposed to encompass these new characteristics with the aim of classifying the data more effectively and reducing the presence of false positives.

To improve the IFPTML-Flaviviridae model, the *D*_*k*_ was expanded from 3 to 30 for every ***c***_***j***_ = 6. The increase in *D*_*k*_ provides new unique characteristics of the compounds that can be used to compare the data in a better way and reduce false positives that may arise due to similarities. The number of combinations *n*^*n*^ allows for a more comprehensive discrimination of the information and, therefore, a more specific classification by having more parameters to evaluate, which helps determine the influence of each descriptor in the system.

The new model uses the 46,518 assays from the IFPTML-Flaviviridae model as the data set. After the LDA analysis, favorable statistical results were obtained for Sp (%), Sn (%), and Ac (%) in training and validation sets. When comparing the IFPTML-Flaviviridae model with the IFPTML-Flaviviridae *D*_*k*_30 model, an increase in the statistical parameters of the *D*_*k*_30 model was observed, with an accuracy Ac (%) increasing from 77 to 79%, Sp (%) from 75.95 to 78.58%, and Sn (%) from 78.88 to 80.06%.

By including the *Flavivirus* genus model, it can be observed that the Sp (%) value in the *Flavivirus* model is higher than that in its counterparts (Flaviviridae models). Thereby, the selection of negative values is done correctly as shown in its classification matrix. When comparing Sn (%), it is evident that the Flaviviridae *D*_*k*_30 model shows better values, indicating that it classifies true values more accurately. This is reflected in the classification matrix results, where the false positives reported in the IFPTML-Flaviviridae *D*_*k*_30 model compared with the IFPTML-*Flavivirus* model are in a smaller proportion, indicating that there is an improving data classification. The accuracy of the IFPTML-Flaviviridae *D*_*k*_30 model improves by 2 points compared with IFPTML-Flaviviridae model. Although the IFPTML-*Flavivirus* model has an accuracy Ac (%) of 82%, the IFPTML-Flaviviridae *D*_*k*_30 model shows better overall results in terms of statistical parameters and classification matrix. Finally, the IFPTML-Flaviviridae *D*_*k*_30 model was selected as the final model (see Table 5).

**Table 5:** Results of the IFPTML-Flaviviridae *D*_*k*_30-LDA Model, Statistical Parameters Sp, Sn and Ac for Training and Validation

sets	param.		scope of test *p*(1) =		
expected stat.		predicted values	***n***_***j***_	***f***(***v***_***ij***_)_**pred**_ = **0**	***f***(***v***_***ij***_)_**pred**_ = **1**
Training Series					
***f***(***v***_***ij***_)_**obs**_ = **0**	Sp (%)	78.58	19,338	15,236	4152
***f***(***v***_***ij***_)_**obs**_ = **1**	Sn (%)	80.06	15,500	3091	12,409
total	Ac (%)	79.23	34,888		
Validation Series					
***f***(***v***_***ij***_)_**obs**_ = **0**	Sp (%)	78.35	6457	5059	1398
***f***(***v***_***ij***_)_**obs**_ = **1**	Sn (%)	80.41	5172	1013	4159
total	Ac (%)	79.27	11,629		

### 3.3 ROC Validation IFPTML-Flaviviridae-LDA Method

**Figure 2:**
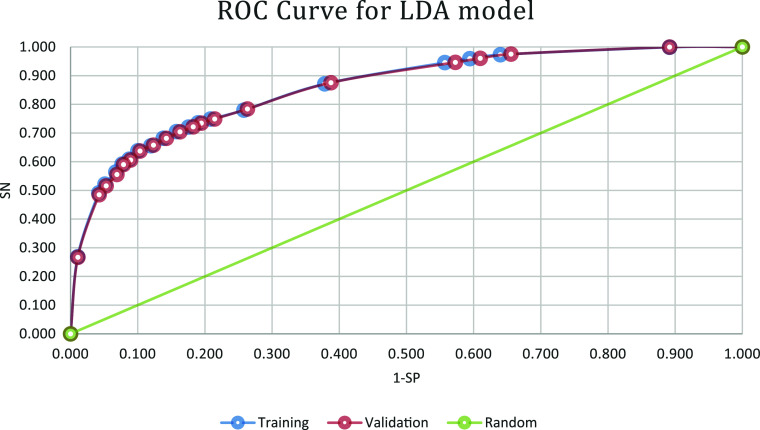
AUCROC curve graph, the diagonal line represents random probability is 0.5, In the graph, it can be observed how the values of probability changes, orange represents the validation set and blue the training set.

The validation method that was used is a AUCROC curve.^47^ This method verifies the reliability of the models by using sensitivity Sn (%) vs precision (1 – Sp (%)), obtaining a AUCROC curve based on this input data. Subsequently, the area under the curve is calculated. The sensitivity and precision values of the training and validation models were used to represent the curve with a layout that changes with the prior probability. In the graph, it can be observed how the values of Sn (%) and (1 – Sp (%)) degrade uniformly as the prior probability changes (see Figure 2).

Within the AUCROC curve graph, the diagonal line represents random probability, where *p*(*v*_*ij*_ = 1) = 0.5. The area under the curve (AUROC) for a random classification model is 0.5. The AUROC value obtained by the IFPTML model is 0.862, indicating that the discrimination is accurately 86.2% and not a random pattern. This type of validation test uses probability based on Bayes’ theorem.^54,55^ Throughout the study, prior probability values were used before applying the theory, allowing the study of the change of Sn (%) and (1 – Sp (%)) over time to obtain an optimal value.

The technique itself is based on the area under the curve, so by performing this ROC curve, the predictive power of the model is obtained. The *R* value of 86.2% indicates that this model allows for deciding which elements are related or not with a high degree of classification.

### 3.4 Comparison of Classification ML Models through Python

Currently, several ML algorithms are used for classification and prediction tasks, each of them with its own and unique characteristics. LDA, random forest (RF), and gradient boosting (GB) are among the most popular methods.^55−57^

The IFPTML-LDA models use the LDA-supervised learning algorithm for classification. This method assumes that the input data follows a Gaussian distribution, and the classes have an equal covariance matrix.^58^ The algorithm finds linear combinations of features that maximize the separation between classes. This methodology is useful when the classes are well-separated.^59^ On the other hand, models based on Random Forest are ensemble learning methods that combine multiple decision trees to make predictions,^60^ They create a collection of decision trees, where each tree is trained on a random subset of data and features.^61^ This learning method can handle both classification and regression tasks. It is known for its ability to handle complex relationships and interactions in data.^62^ Finally, Gradient Boosting combines multiple weak learners (usually decision trees) to create a strong learner. It builds a model in an iterative manner, where each new model focuses on correcting the mistakes made by previous models.^63^ Also, it is known for its high predictive accuracy and ability to handle complex data sets, and it works well for both classification and regression tasks.^64^

The different learning methods used to compare the ML techniques have pros and cons, as mentioned earlier in this section. The LDA methodology is more suitable for well-separated classes and dimensionality reduction. RF is effective in handling complex relationships and providing important feature measures. GB is known for its high predictive accuracy and ability to handle complex data sets. The complete ML methods used are presented bellow (See Table 6).

**Table 6:** IFPTML-Flaviviridae *D*_*k*_30 Model Comparation of the Most Used ML Methods in Classification and Regression Task^a^

model comparation by 10-fold CV									
model		accuracy	AUC	recall	prec.	F1	kappa	MCC	TT (s)
lightgbm	light gradient boosting machine	0.784	0.891	0.749	0.761	0.749	0.561	0.568	8.812
xgboost	extreme gradient boosting	0.783	0.886	0.749	0.759	0.749	0.559	0.565	4.896
ridge	ridge classifier	0.783	NA	0.693	0.785	0.729	0.552	0.561	0.736
lda	linear discriminant analysis	0.782	0.872	0.697	0.781	0.729	0.551	0.559	2.654
gbc	gradient boosting classifier	0.781	0.882	0.713	0.772	0.733	0.550	0.559	57.433
ada	ada boost classifier	0.774	0.868	0.691	0.771	0.720	0.534	0.544	11.047
lr	logistic regression	0.771	0.855	0.701	0.761	0.724	0.531	0.538	14.002
rf	random forest classifier	0.770	0.852	0.724	0.749	0.731	0.531	0.537	16.707
dt	decision tree classifier	0.766	0.812	0.716	0.748	0.726	0.523	0.529	3.626
et	extra trees classifier	0.765	0.833	0.706	0.749	0.722	0.520	0.526	13.657
knn	K neighbors classifier	0.731	0.788	0.706	0.691	0.696	0.455	0.458	4.982
svm	SVM—linear kernel	0.628	NA	0.561	0.634	0.553	0.244	0.266	5.079
qda	quadratic discriminant analysis	0.612	0.673	0.779	0.547	0.638	0.247	0.272	1.656
nb	naive bayes	0.609	0.667	0.609	0.554	0.578	0.216	0.218	0.254
dummy	dummy classifier	0.556	0.500	0.000	0.000	0.000	0.000	0.000	1.209

a The Pycaret library was used to build the ML models and some of the algorithms such as ridge and SVM do not support “predict_proba”. In those cases, the AUC value is shown as “NA”.

The model was tuned with 5- and 10-folds. Although the values are quite similar, the base model shows the best performances, thus, it was selected as the best model (see Table 7).

**Table 7:** Performance Metrics of IFPTML-Flaviviridae *D*_*k*_30 Model with 5- and 10-Folds

	folds	accuracy	AUC	recall	prec.	F1	kappa	MCC
base	10	0.78	0.89	0.75	0.76	0.75	0.56	0.57
tuned	10	0.78	0.89	0.71	0.78	0.73	0.55	0.56
	5	0.78	0.88	0.73	0.77	0.74	0.55	0.56

The IFPTML-Flaviviridae *D*_*k*_30 model was evaluated using the validation set, and the LightGBM model showed the best overall performance metrics (see Table 8). Therefore, the optimization step was performed using this model.

**Table 8:** Light Gradient Boosting Machine Presents the Best Results Using the Flaviviridae *D*_*k*_30data Set

model	accuracy	AUC	recall	prec.	F1	kappa	MCC
light gradient boosting machine	0.83	0.92	0.79	0.82	0.80	0.65	0.65

**Figure 3:**
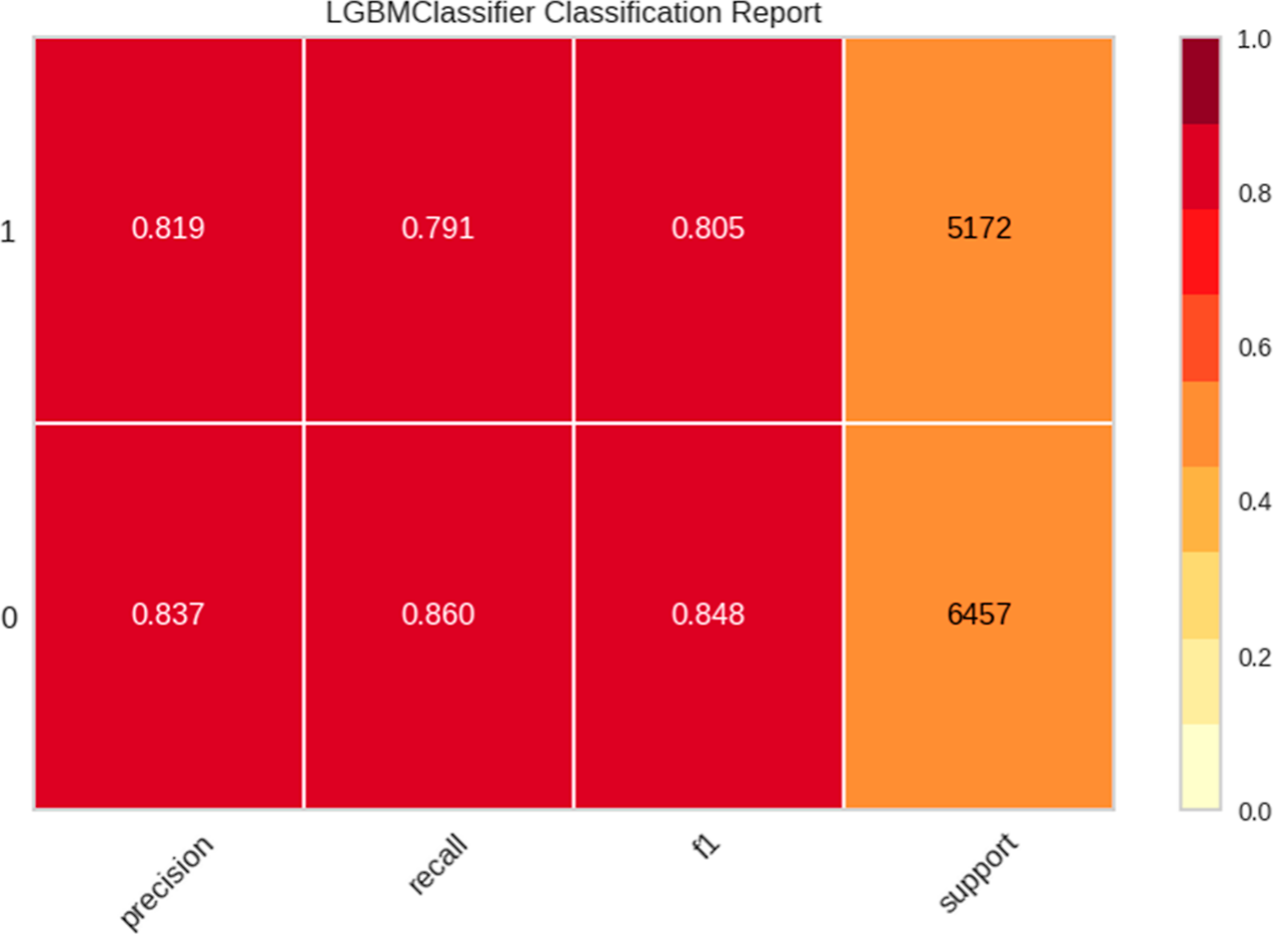
LGBM Classification Report of the IFPTML-Flaviviridae *D*_*k*_30 model—validation data set.

The classification report is used as an evaluation method to measure the performance of the classification model. This report provides information on the performance of the model for each class in terms of accuracy, recall, F1, etc.^65^ The LGBM model presents a precision value of 0.82. This value measures the proportion of correctly predicted positive instances out of all instances predicted as positive. This indicates the reliability of the model’s positive predictions; a higher value means fewer false positives. The recall value of 0.79 represents the sensitivity, or true positive rate. This shows the proportion of correctly predicted positive instances out of all actual positive instances, indicating how well the model identifies positive instances. A higher value means fewer false negatives.^66^ The F1 value of 0.80 is a harmonic mean between precision and recall, providing a single metric that balances both. Figure 3 shows the classification report of the IFPTML-Flaviviridae *D*_*k*_30—LGBM model.

**Figure 4:**
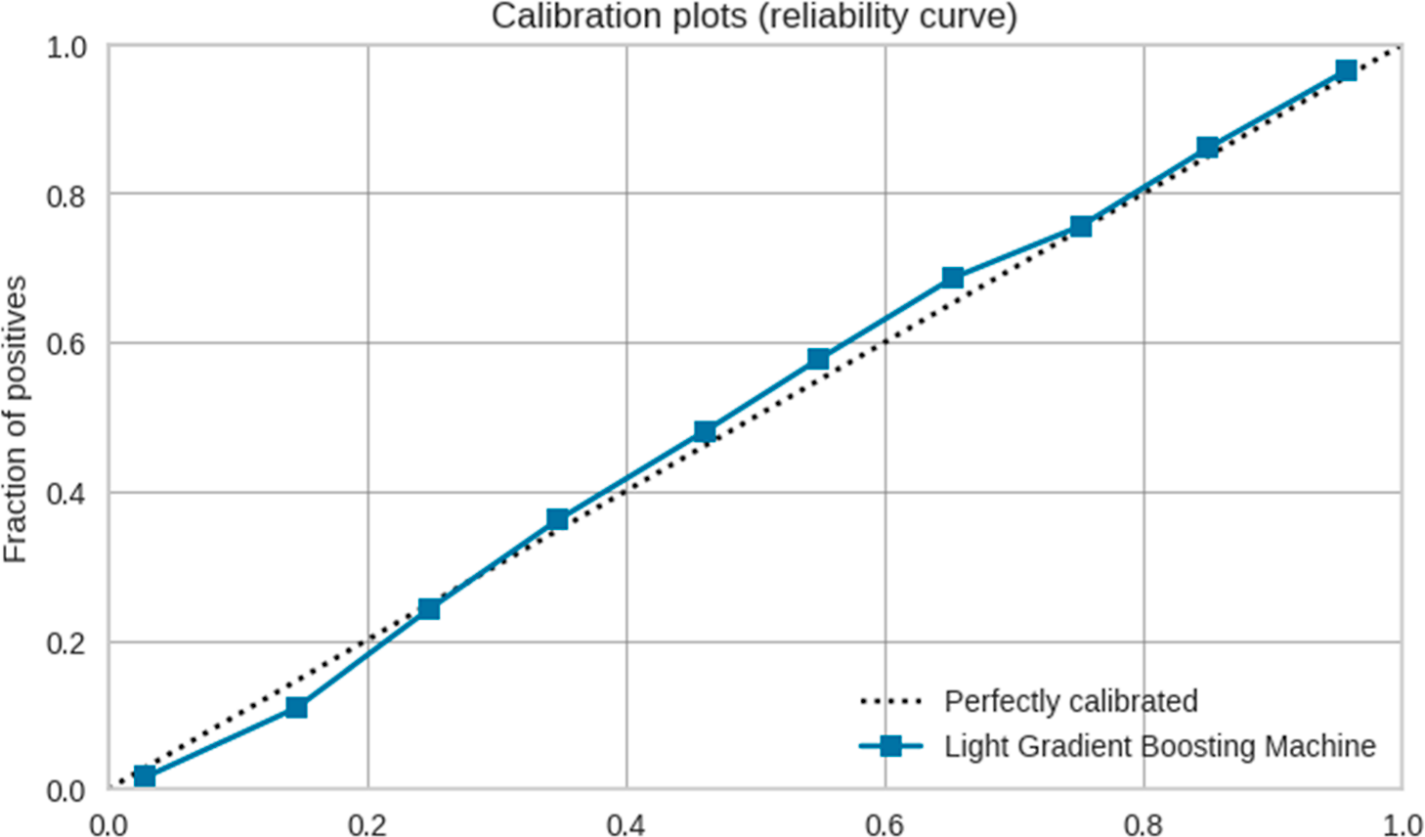
Calibration plots of the IFPTML-Flaviviridae *D*_*k*_30—LGBM.

The reliability curve was used to assess the calibration of the classification model. This type of calibration plot helps determine whether the predicted probabilities are well-calibrated and provides reliable estimates of true probabilities. Figure 4 presents the calibration plot using LGBM model. The curve closely follows the diagonal line, suggesting good calibration of the model. No overconfidence or under confidence was identified in the curve.

The calibration plots provide information on the agreement between the probabilities predicted by the model and the actual probabilities. An accuracy value of 0.83 means that if the model predicts probabilities for a given class, the actual probability of that class should be close to that value.^24,25^

**Figure 5:**
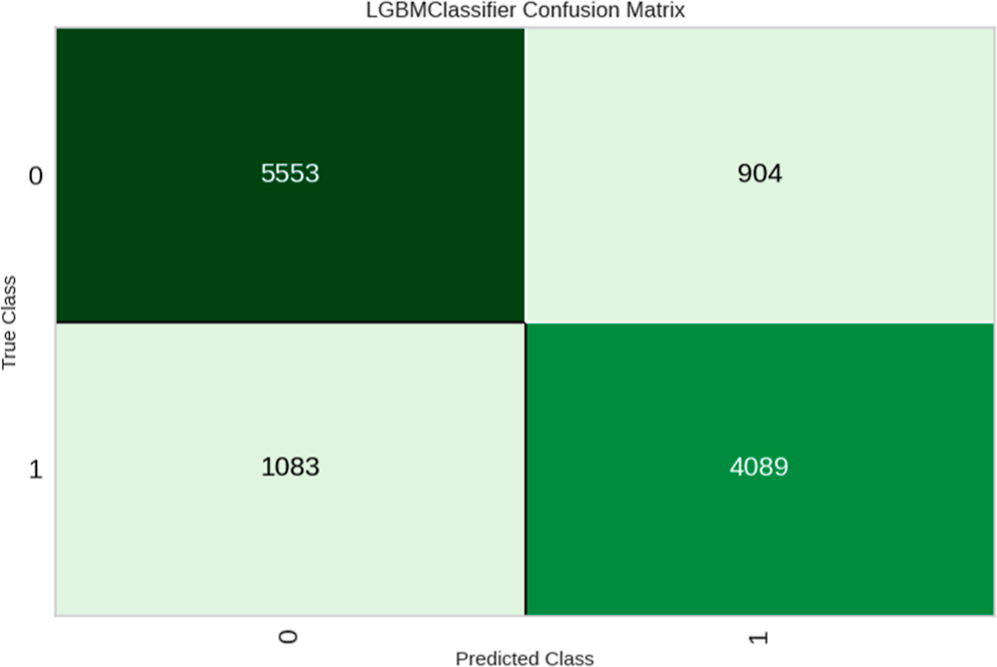
LGBM confusion matrix using the validation data set of Flaviviridae *D*_*k*_30.

As mentioned in the previous method analysis, the variation between the members of the Flaviviridae family and the limited amount of available data for the assays can result in the presence of false positives. During the expansion of *D*_*k*_ in the IFPTML-LDA models, a reduction in the number of false positives was achieved. Using the same data set in the LGBM model, another reduction in false positives was achieved, resulting in an improvement in the reliability of the model, with the number of false positives decreasing from 1398 to 904 (0:1) (see Figure 5). A comparison between the *D*_*k*_30-LDA and *D*_*k*_30-LGBM models showed a reduction of 4.25% in false positives.

**Figure 6:**
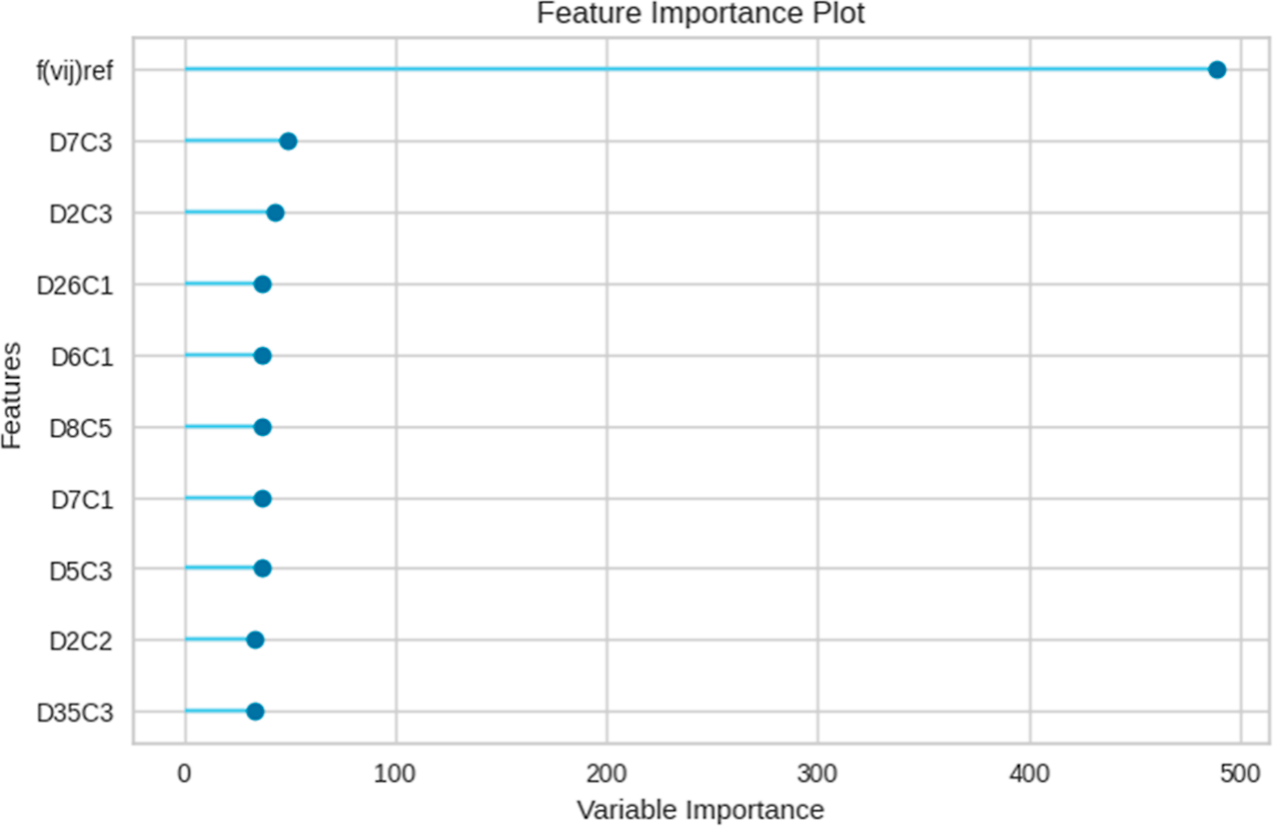
Feature Importance Plot of the IFPTML-Flaviviridae *D*_*k*_30-LGBM model.

The feature importance plot is a graphical representation of the importance of each feature in a ML model. This helps identify the features that have the most significant impact on the model’s predictions. Figure 6 presents a feature importance plot where the most influential features are ranked. Each feature is assigned a score that represents its importance. The higher the score, the more influential the feature is in making predictions.^36^ In tree-based models such as gradient boosting models, the importance is calculated based on the number of times a feature is used to split the data across all trees. However, its importance is not a definitive measure of causality. It only indicates the relative importance of the features within the context of the model.

**Figure 7:**
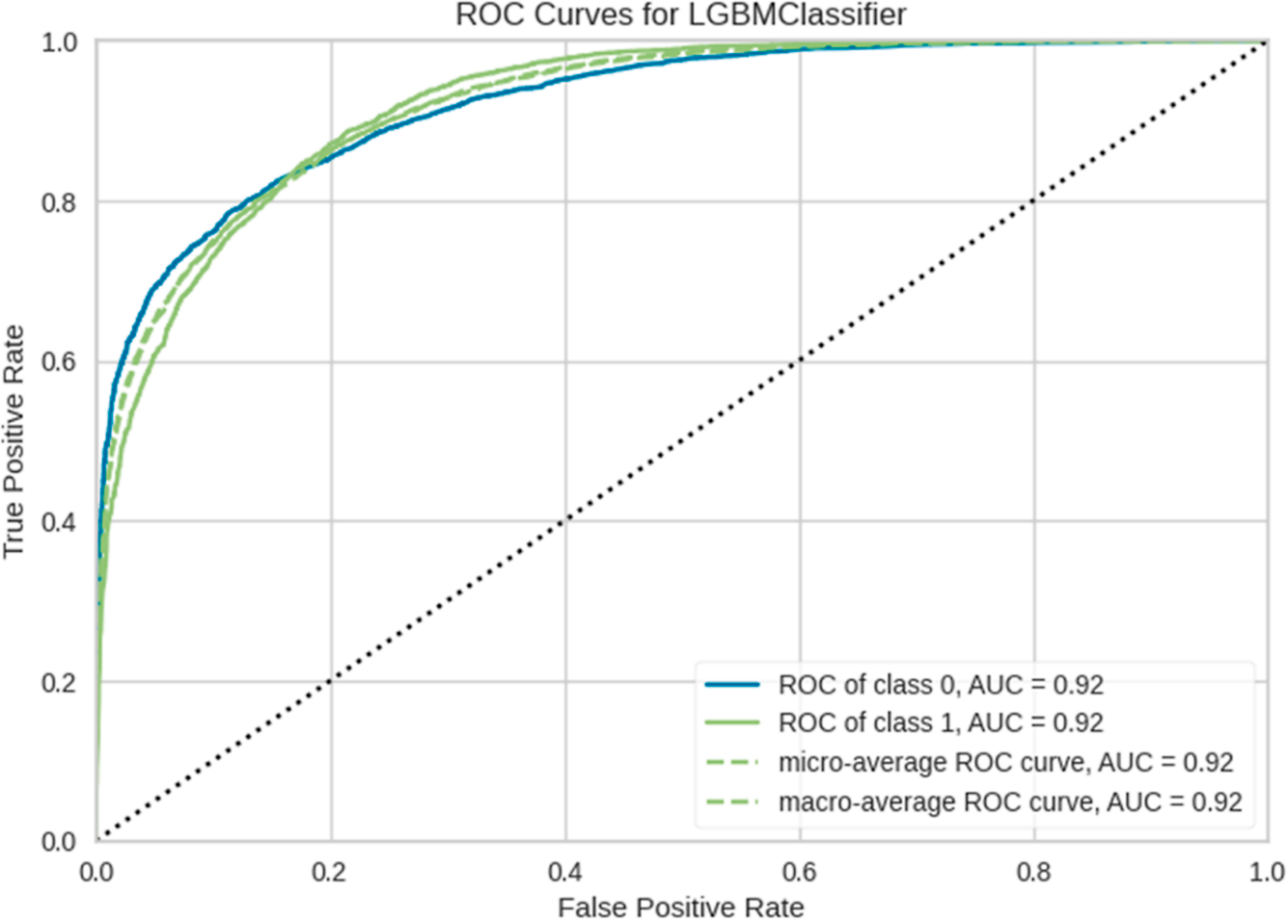
AUCROC curve for the LGBM classifier of IFPTML-Flaviviridae *D*_*k*_30 model.

A graphical representation of the AUCROC curve illustrates the performance of the binary classifier system. As shown in Figure 7, the LGBM classifier presents an AUCROC curve of the IFPTML-LGBM model, which provides a visual representation of the trade-off between the true and false positive rates. A good classifier will have a curve closer to the top-left corner of the plot.

The AUCROC value of 0.92 can be interpreted as the probability that the classifier will rank a randomly chosen positive instance higher than a randomly chosen negative instance. A higher AUCROC indicates better performance.^47^ Comparing the AUCROC of the LDA model vs LGBM model, it shows an increase of 6%, indicating an upgrade in the classification model compared with its older version.

Finally, this representation indicates that the data are related and that the assay conditions can be tested in several assays of these viral diseases. (see Supporting Information DATASET.xlsx for more information).

## 4 Conclusion

The results obtained throughout all the studies present a model capable of being incorporated into drug development research against diseases related to the Flaviviridae family. In summary, the IFPTML models are capable of handling, classifying, and processing large data sets with high specificity. The effectiveness of the models, as indicated by the statistical parameters, corroborates their effectiveness against this type of data.

The IFPTML-LGBM model presents the most solid results, with an accuracy of 83% in the validation sets. The model also achieves a 92% AUCROC value, indicating that it classifies true positives with high accuracy. Through feature enrichment with more assay information, the classification system was improved, upgrading the classification method by 6% compared to previous versions of the models.

The selection of ML methods for classification should be based on the type of data and groups of data that the system is going to use. The model is capable of processing and identifying new candidate compounds that may have biological activity against Flaviviridae family diseases.

Finally, these results mark a starting point for the development of new techniques based on ML models that can help in the discovery of novel drugs and treatments against diseases of the Flaviviridae family.

## Data Availability

The data set and code of the model are publicly available under the MIT license on GitHub in the following link: https://github.com/Aruize/IF.PTML-Flaviviridae. The Supporting Information including all the data used in this paper. Not proprietary data is reported on this work. Materials and Methods describe all the theory at a level that allows a person skilled in the art could implement the method.

## Supporting Information Available

ChEMBL data set used to train and validate the model, compounds codes, SMILE codes, preclinical assay conditions, observed values, predicted classifications, probabilities, etc. (File DATASET.xlsx) (XLSX)
